# The Pharmacogenomics of Bipolar Disorder study (PGBD): identification of genes for lithium response in a prospective sample

**DOI:** 10.1186/s12888-016-0732-x

**Published:** 2016-05-05

**Authors:** Ketil J. Oedegaard, Martin Alda, Anit Anand, Ole A. Andreassen, Yokesh Balaraman, Wade H. Berrettini, Abesh Bhattacharjee, Kristen J. Brennand, Katherine E. Burdick, Joseph R. Calabrese, Cynthia V. Calkin, Ana Claasen, William H. Coryell, David Craig, Anna DeModena, Mark Frye, Fred H. Gage, Keming Gao, Julie Garnham, Elliot Gershon, Petter Jakobsen, Susan G. Leckband, Michael J. McCarthy, Melvin G. McInnis, Adam X. Maihofer, Jerome Mertens, Gunnar Morken, Caroline M. Nievergelt, John Nurnberger, Son Pham, Helle Schoeyen, Tatyana Shekhtman, Paul D. Shilling, Szabolcs Szelinger, Bruce Tarwater, Jun Yao, Peter P. Zandi, John R. Kelsoe

**Affiliations:** Department of Clinical Medicine, Section for Psychiatry, Faculty of Medicine, University of Bergen, Bergen, Norway; Division of Psychiatry, Haukeland University Hospital, Bergen, Norway; Department of Psychiatry, Dalhousie University, Halifax, Canada; NORMENT, KB Jebsen Centre for Psychosis Research, Division of Mental Health and Addiction, Oslo University Hospital, and Institute of Clinical Medicine, University of Oslo, Oslo, Norway; Department of Psychiatry, University of Pennsylvania, Philadelphia, PA USA; Department of Psychiatry, University of California San Diego, La Jolla, CA 92093 USA; Department of Psychiatry, VA San Diego Healthcare System, La Jolla, CA USA; Department of Psychiatry, Icahn School of Medicine at Mt Sinai, 1 Gustave L. Levy Pl, New York, NY 10029 USA; Department of Neuroscience, Icahn School of Medicine at Mt Sinai, 1 Gustave L. Levy Pl, New York, NY 10029 USA; Department of Psychiatry, Case Western Reserve University School of Medicine, Cleveland, OH USA; Department of Psychiatry, University of Iowa, Iowa City, IA USA; Neurogenomics Division, The Translational Genomics Research Institute, Phoenix, AZ USA; Department of Psychiatry, The Mayo Clinic, Rochester, MN USA; Laboratory of Genetics, The Salk Institute for Biological Studies, La Jolla, CA USA; Department of Psychiatry, University of Chicago, Chicago, IL USA; Department of Psychiatry, University of Michigan, Ann Arbor, MI USA; Department of Psychiatry, Indiana University, Indianapolis, IN USA; Department of Psychiatry, Johns Hopkins University, Baltimore, MD USA; St. Olav University Hospital of Trondheim and Department of Neuroscience, Faculty of Medicine, Norwegian University of Science and Technology, Trondheim, Norway; University of Bergen, Faculty of Medicine and Dentistry, Division of Psychiatry, Stavanger University Hospital, Stavanger, Norway

**Keywords:** Bipolar disorder, Lithium, Mood stabilizer, Pharmacogenetics, GWAS, Prospective trial, Personalized medicine, Precision medicine

## Abstract

**Background:**

Bipolar disorder is a serious and common psychiatric disorder characterized by manic and depressive mood switches and a relapsing and remitting course. The cornerstone of clinical management is stabilization and prophylaxis using mood-stabilizing medications to reduce both manic and depressive symptoms. Lithium remains the gold standard of treatment with the strongest data for both efficacy and suicide prevention. However, many patients do not respond to this medication, and clinically there is a great need for tools to aid the clinician in selecting the correct treatment. Large genome wide association studies (GWAS) investigating retrospectively the effect of lithium response are in the pipeline; however, few large prospective studies on genetic predictors to of lithium response have yet been conducted. The purpose of this project is to identify genes that are associated with lithium response in a large prospective cohort of bipolar patients and to better understand the mechanism of action of lithium and the variation in the genome that influences clinical response.

**Methods/Design:**

This study is an 11-site prospective non-randomized open trial of lithium designed to ascertain a cohort of 700 subjects with bipolar I disorder who experience protocol-defined relapse prevention as a result of treatment with lithium monotherapy. All patients will be diagnosed using the Diagnostic Interview for Genetic Studies (DIGS) and will then enter a 2-year follow-up period on lithium monotherapy if and when they exhibit a score of 1 (normal, not ill), 2 (minimally ill) or 3 (mildly ill) on the Clinical Global Impressions of Severity Scale for Bipolar Disorder (CGI-S-BP Overall Bipolar Illness) for 4 of the 5 preceding weeks. Lithium will be titrated as clinically appropriate, not to exceed serum levels of 1.2 mEq/L. The sample will be evaluated longitudinally using a wide range of clinical scales, cognitive assessments and laboratory tests. On relapse, patients will be discontinued or crossed-over to treatment with valproic acid (VPA) or treatment as usual (TAU). Relapse is defined as a DSM-IV manic, major depressive or mixed episode or if the treating physician decides a change in medication is clinically necessary. The sample will be genotyped for GWAS. The outcome for lithium response will be analyzed as a time to event, where the event is defined as clinical relapse, using a Cox Proportional Hazards model. Positive single nucleotide polymorphisms (SNPs) from past genetic retrospective studies of lithium response, the Consortium on Lithium Genetics (ConLiGen), will be tested in this prospective study sample; a meta-analysis of these samples will then be performed. Finally, neurons will be derived from pluripotent stem cells from lithium responders and non-responders and tested in vivo for response to lithium by gene expression studies. SNPs in genes identified in these cellular studies will also be tested for association to response.

**Discussion:**

Lithium is an extraordinarily important therapeutic drug in the clinical management of patients suffering from bipolar disorder. However, a significant proportion of patients, 30–40 %, fail to respond, and there is currently no method to identify the good lithium responders before initiation of treatment. Converging evidence suggests that genetic factors play a strong role in the variation of response to lithium, but only a few genes have been tested and the samples have largely been retrospective or quite small. The current study will collect an entirely unique sample of 700 patients with bipolar disorder to be stabilized on lithium monotherapy and followed for up to 2 years. This study will produce useful information to improve the understanding of the mechanism of action of lithium and will add to the development of a method to predict individual response to lithium, thereby accelerating recovery and reducing suffering and cost.

**Trial registration:**

ClinicalTrials.gov

Identifier: NCT01272531

Registered: January 6, 2011

## Background

### Clinical presentation and treatment of bipolar disorder

Bipolar disorder is a major psychiatric disorder described by alternations between the extreme mood states of mania and depression and characterized by episodic relapses and chronic disability [1]. Bipolar disorder affects approximately 1 % of the population worldwide. It is a major cause of hospitalizations and healthcare expenditures, as well as suicide [2].

While there is no cure for bipolar disorder, it is a highly treatable and manageable illness. After an accurate diagnosis, most people (80–90 %) can achieve substantial stabilization of their mood and related symptoms with proper treatment. Medications known as mood stabilizers are usually prescribed to help control bipolar disorder. These are used both in the acute treatment of episodes and as maintenance treatment to prevent new episodes. The medications are in general effective in both the manic and depressive phases of the illness, hence the term mood stabilizer. However, in practice, these medications tend to be more effective against acute mania than acute depression.

The action of lithium as a mood stabilizer was first identified by John Cade [3]. Since that time, it has remained the standard against which other medications are compared [4]. Several different types of mood stabilizers are available in addition to lithium. These include some, but not all, anticonvulsants such as valproate, carbamazepine, and lamotrigine [5]. Atypical antipsychotics such as olanzapine, risperidone, aripiprizole and quetiapine also have demonstrated efficacy as mood stabilizers [5].

Though there are now many effective mood stabilizers, lithium has remained the treatment of choice for bipolar disorder. Although lithium is effective in the majority of patients, 30–40 % fail to respond. Furthermore, a substantial number of patients cannot be maintained on lithium because of intolerable side effects such as weight gain, acne, thyroid suppression, and renal impairment. In recent years, clinical data have demonstrated that valproic acid, lamotrigine, olanzapine and other medications may have comparable efficacy to lithium [6–8]. Furthermore, these novel mood stabilizers have been thought to have a more benign side effect profile and to be better tolerated by patients. For these reasons, valproic acid, lamotrigine, olanzapine, aripiprizole, and quetiapine have begun to supplant lithium as the most frequently prescribed medication for bipolar disorder. However, the cost of treatment with these newer agents is in excess of 10 times higher than that of lithium ($60/mo for valproic acid vs. $15/mo for olanzapine vs. $1/mo for lithium). Also, other recent clinical studies, such as the BALANCE trial, have demonstrated superior overall efficacy of lithium for relapse prevention in comparison to valproate [9, 10]. Furthermore, a subset of patients do extremely well on lithium and have excellent control of episodes with few, if any, side effects.

As detailed below, a variety of data argue that good lithium responders constitute a clinically and genetically distinct group. As there is currently no method to identify this subset of lithium responders, lithium is being used less frequently despite the fact that many patients would respond very well to it. Furthermore, many patients who might have been excellent lithium responders are never able to benefit from lithium treatment because lithium is never tried. Development of a method to predict individual response to lithium before initiation of treatment would, therefore, be of great benefit to clinicians in making prescribing decisions and in accelerating recovery and reducing cost.

### Predictors of response

A variety of clinical and biological predictors, as well as genetic data, suggest that lithium-responsive bipolar disorder may comprise a mechanistically distinct form of illness.

#### Clinical predictors

A recent scholarly review of the various predictors of good and poor outcome following long-term treatment with lithium proposes a useful nomenclature that classifies these predictors according to category [11]. Psychopathological predictors of good response include initial good response within 6–12 months (highly reliable), classic euphoric/elated mania, positive family history of bipolar disorder with a good response to lithium in particular, the absence of a personality disorder, bipolar I disorder (as opposed to type II), the mania-depression-euthymic pattern of illness course, the presence of melancholic features during depressive episodes, and early onset of lithium therapy. Predictors of poor response in this category included mixed episodes (highly reliable), rapid cycling, mood incongruent psychotic symptoms, onset before the age of 18, high numbers of lifetime mood episodes, and the depression-mania-euthymia pattern of illness course. The most highly replicated environmental predictor is that of being single, not married.

#### Biological predictors

Biological predictors of good response to lithium include high RBC/plasma-lithium ratio (highly controversial), higher platelet serotonin-induced calcium mobilization, and high rate of red blood cell membrane phospholipids in lithium intracellular transport. Neurophysiologic predictors of good response include brain lithium concentrations above 0.2 mEq/L when measured by 7Li-MRS, decreased cerebral intracellular pH, white matter hyperintensity at (31)P-MRS, and high intensity of loudness dependent auditory evoked potentials. Neurophysiologic predictors of poor response have included epileptiform anomalies with diffuse theta waves on EEG and decreased cerebral phosphocreatinine levels at (31)P-MRS. Genetic predictors of good response include lower inositol monophosphatase mRNA expression and a high frequency of phospholipase C isoenzyme gamma-1. Genetic predictors of poor response include homozygotic forms of the short allele of the serotonin transporter gene (5-HTT), the presence of the A/A subtype of tryptophan hydroxylase (TPH), and a high frequency of human leukocyte antigens type A3 (HLA-A3).

### Mechanism of action of lithium

A major challenge in the discovery of genes for mood stabilizer response is the lack of understanding of the pathophysiology of the disease and the mechanism of action of the existing medications. Lithium, as the oldest mood stabilizer, has received the most attention in terms of its mechanism of action. A variety of protein targets and signal transduction processes have been reported to be inhibited by lithium. Therefore, the challenge has been determining which of these mechanisms are therapeutically important. Some of the earliest studies reported that cAMP generation in response to adrenaline was inhibited by lithium [12]. Early on the theme began to emerge that inhibition of both sides of systems in balance led to an overall stabilization and reduced biochemical swings that might in turn underlie behavioral swings. One of the first such observations was that cAMP and cGMP production by norepinephrine and epinephrine, respectively, were both inhibited by lithium [13]. Lithium was also later reported by the same group to inhibit both adrenergic- and cholinergic-induced GTP binding in rat brain [14]. G proteins as a target of lithium received further support with the observation that lithium inhibits expression of the gene for Gαi [15] and that it inhibits the action of Gαs [16].

The inositol signaling pathway has also been a major focus of lithium studies. In response to receptor stimulation, phospholipase C (PLC) breaks down phosphotidyl inositol 4,5-biphosphate (PIP2) to inositol 1,4,5-triphosphate (IP3) and diacylglycerol (DAG). These products act as intracellular signaling molecules triggering the release of calcium from intracellular stores and activating protein kinase C (PKC). Inositol monophosphatase (IMPase) and inositol polyphosphatase (IPPase) then dephosphorylate IP3 and IP2 and recycle free inositol to the membrane. Lithium has been observed to inhibit IMP and IPP, leading to the hypothesis that lithium treatment leads to a depletion of free inositol, thereby inhibiting this signaling pathway [17, 18].

Protein kinase C is a downstream target of phosphoinositide signaling that has received extensive attention. PKC activity has been shown to be reduced by lithium [19] and has been reported to be elevated in mania [20]. At doses adequate to inhibit PKC, tamoxifen has been shown to have anti-manic properties clinically [21]. Lithium also reduces the levels of a major substrate of PKC, myristoylated alanine-rich C kinase substrate (MARCKS) [22].

The profound effects of lithium on morphogenesis in Drosophila led to a novel hypothesis of lithium’s mechanism of action [23]. Wnt signaling proteins bind to transmembrane *frizzled* receptors and inhibit glycogen synthase kinase 3β (GSK3β) via *disheveled*. This inhibition in turn promotes the stabilization of β-catenin. Lithium was shown to inhibit GSK3β and thereby mimic the effect of wnt signaling [24]. Lastly, the most recent and novel proposed mechanism of action of lithium involves neuroprotection. Lithium has been shown to protect neuronal cultures from glutamate-induced apoptosis [25]. Lithium has also been shown to increase expression of the anti-apoptotic protein, bcl-2 [26]. The neuroprotective effect of lithium may also be mediated by stimulating the phosphorylation of Akt-1 by phosphoinositide kinase (PI3-K), which in turn phosphorylates GSK3β [27]. This process may inhibit the pro-apoptotic action of GSK3β.

### Lithium-mediated gene expression

There are likely hundreds if not thousands of genes that are modulated in some way by lithium. Data have been emerging for the past several years that clearly demonstrate lithium-mediated gene expression changes. Bosetti et al. reported changes in the expression of a variety of genes in response to lithium, including those from signal transduction pathways [28]. The study of a larger gene array with 39,000 transcripts in mice [29] reported 4,474 genes with nominally significant expression changes and 121 with a stringent Bonferroni correction. There were 13 genes affiliated with phosphotidylinositol metabolism, again consistent with the cell signaling pathway hypothesis. One of the more comprehensive studies reported to date used a human neuronal cell line (SK-N-AS), and they reported 347 upregulated and 324 downregulated genes, from 3 primary pathways: the mitogen-activated protein kinase (MAPK) signaling pathway, an actin cytoskeleton pathway and a calcium signaling pathway [30].

Expression patterns of micro RNAs (miRNA) in the hippocampus of rats have shown that lithium and valproate both decreased the expression of several miRNAs considered candidates for bipolar disorder; including the pathways involving Wnt/βcatenin [31]. A study of miRNA in lymphoblastoid cell lines found increased expression when exposed to lithium, with a clear variation in expression patterns with time, reflecting the dynamic biological system wherein gene expression is unlikely to be static [32].

### Genetics of mood stabilizer response

There is much evidence to suggest that genetic factors play a strong role in the variation in response to lithium. Several studies have indicated that lithium-responsive patients are more likely to have a stronger family history of bipolar disorder than lithium non-responders [33, 34]. Mendlewicz et al. have reported better lithium prophylaxis in concordant bipolar twin pairs than in discordant pairs [35]. Grof et al. have shown that affected relatives of lithium-responsive bipolar probands are more likely to also be lithium responsive than the relatives of non-lithium-responsive probands [36]. Based on the idea that lithium-responsive bipolar disorder is a distinct genetic subtype, several studies have focused on this phenotype for mapping genes for bipolar disorder. Turecki et al. have reported a genome scan of such families with significant evidence of linkage to a locus on 15q [37]. Two studies have reported association of the gene PLCG1 to lithium-responsive bipolar disorder [38, 39]. Two specific genes have been reported to be associated with response to lithium in a comparison of responders and non-responders: the serotonin transporter [40] and inositol polyphophatase (INPP1) [41]. One GWAS has been performed on a subset of the STEP-BD sample including 359 bipolar I or bipolar II patients [42]. The strongest region was on 10p15 (*p* = 10-7), and the gene GRIA2 also showed some evidence for association.

### Research objectives

#### Aim

The long-term goal of this project is to better understand the mechanism of action of lithium and variation in the genome that influences response. Practically, the hope is that this understanding may lead to a DNA test that would aid physicians in selecting mood stabilizers. It also may add to our understanding of bipolar disorder by distinguishing a genetically distinct form.

#### Specific aims

1. A prospective trial of mood stabilizer response will be conducted at a group of 11 closely collaborating sites.
  - The study will have an algorithmic design. All patients will be started on lithium. Those that fail lithium will be crossed over to valproic acid (VPA). Those that fail VPA will be crossed over to a standardized treatment as usual (TAU) arm.
  - The study will be designed to identify genes for relapse prevention. All patients will be stabilized on lithium monotherapy and then enter the maintenance phase where they will be followed for 2 years or until relapse. On relapse, they will be discontinued or crossed over as appropriate.
  - The prospective sample will be genotyped for GWAS. Positive SNPs from the Consortium on Lithium Genetics (ConLiGen) will first be selectively tested to control multiple comparisons. Following this, a complete GWAS on the prospective sample will be performed as well as a meta-analysis with ConLiGen.
2. Mechanistic studies of lithium response will be conducted as part of the project.
  - Skin biopsies will be collected on 100 patients from the prospective study. Fibroblasts from 10 lithium responders and 10 non-responders will be reprogrammed to induced pluripotent stem cells (iPSCs).
  - iPSCs will be differentiated into neural progenitor cells (NPCs) and then into mature neurons.
  - Pathways will be identified that are altered in lithium responsive vs. non-responsive NPCs and mature neurons. Cells will be treated with and without lithium and RNA-seq will be conducted. Genes whose expression is altered by lithium in lithium responders and not non-responders will be identified as possibly involved in lithium response. SNPs in these genes will be tested for association with response in the human prospective data. Similarly, genes associated with lithium response in the human studies will be tested for change in the cell culture models. Network and pathway analysis will be applied to the expression data. Expression in iPSC-derived neurons will be used to predict networks of related genes. These networks will be tested as groups of genes in the human association data.
  - Specific neuronal deficits will be sought in patient neurons in vitro. Expression profiling results will guide our exploration of cellular phenotypes.

## Methods and design

This study is an 11-site prospective, non-randomized open trial of lithium designed to ascertain a cohort of subjects with bipolar I disorder who experience protocol-defined relapse prevention as a result of treatment with lithium monotherapy. Prior to entry into the 1-month Observation Lithium Monotherapy Phase, three routes of entry will be permitted.

1. Lithium Naïve Patients (LNP): LNPs will enter a 3-month Open Stabilization Phase designed to prospectively demonstrate response to lithium either as a component of Treatment As Usual (TAU) or monotherapy before entry into the 1-month Observation Lithium Monotherapy Phase. LNPs will enter the 1-month Observation Lithium Monotherapy Phase if and when they exhibit a score of 1 (normal, not ill) or 2 (minimally ill) on the Clinical Global Impressions of Severity Scale for Bipolar Disorder (CGI-S-BP Overall Bipolar Illness).
2. Currently Lithium-treated Patients (CLTP): CLTPs on lithium monotherapy will enter the 1-month Observation Lithium Monotherapy Phase when they exhibit a score of 1, 2 or 3 on the CGI-S-BP Overall. For CLTPs as a component of TAU, they will spend up to 3 months during the Open Stabilization Phase slowly weaning off all other psychotropic medications. Once lithium monotherapy is achieved and a score of 1, 2 or 3 on the CBI-S-BP Overall is observed, they will immediately enter the 1-month Observation Lithium Monotherapy Phase.
3. Inadequate Past Lithium-treated Patients (IPLT): IPLTs currently not taking lithium but believed to have had an inadequate past trial of lithium, either due to inadequate dose, blood level, duration, or adherence to treatment, will enter the 3-month Open Stabilization Phase to prospectively demonstrate response to an adequate trial of lithium (either as a component of TAU or monotherapy). IPLTs will enter the 1-month Observation Lithium Monotherapy Phase if and when they exhibit a score of 1 (normal, not ill) or 2 (minimally ill) on the CGI-S-BP.

### Three-month open stabilization phase and One-month observation phase

Lithium, either as a component of guideline-driven adjunctive treatment or monotherapy, will be permitted during the Open Stabilization Phase as clinically indicated. Patients receiving combination therapy will be carefully transitioned to lithium monotherapy as clinically appropriate. During the 1-month Observation Lithium Monotherapy Phase, the CGI-S-BP Overall will be used to describe stability (defined as 4 out of 5 contiguous weeks of a score of 1 or 2), but the study will also assess other relevant outcomes, including depression symptom severity (Montgomery Asberg Depression Rating Scale-MADRS), mania symptom-severity (Young Mania Rating Scale-YMRS), quality of life (Quality of Life Enjoyment and Satisfaction Questionnaire-Q-LES-Q), suicidal behaviors, etc. Following the successful completion of a 3-month Stabilization Phase (if necessary) and a 1-month Observation Lithium Monotherapy Phase, patients meeting ‘a priori’ criteria for inclusion in the Relapse Prevention Phase will be studied for up to 24 months.

### Participants

Inclusion Criteria: 1) Any phase of bipolar I disorder, including depressive, manic, hypomanic, mixed, or baseline/euthymic/not symptomatic; 2) LNPs and IPLTs(as meant?) will be required to have had at least one affective episode in the last 12 months meeting DSM-IV criteria, but CLTPs will be exempted from this criterion if they present without any history of mood episodes meeting DSM-IV criteria in the last 6 months; 3) both outpatients and inpatients will be permitted to enroll into this study; 4) they must be able to give informed consent, in the judgment of the investigator; 5) age greater than or equal to 18 years; 6) women of child-bearing potential agree to inform their doctor at the earliest possible time of their plans to conceive, to use adequate contraception (e.g. oral contraceptives, intrauterine device, barrier methods, or total abstinence from intercourse), and to understand the risks of lithium to the fetus and infant. Depo Provera is acceptable if it is started 3 months prior to enrollment; 7) currently symptomatic, as defined as a CGI-S-BP-Overall of greater than or equal to 3 (mild severity), unless the patient enters the study already stable on lithium monotherapy.

Exclusion Criteria: 1) Unwilling or unable to comply with study requirements; 2) renal impairment (serum creatinine >1.5 mg/dL); 3) thyroid stimulating hormone (TSH) level over >20 % above the upper normal limit (participants maintained on thyroid medication must be euthyroid for at least 3 months before Visit 1; 4) other contraindications to lithium, as will be defined in the medication manual; 5) currently in crisis such that inpatient hospitalization or other crisis management should take priority; 6) although we have not excluded all subjects meeting criteria for alcohol/drug dependence, those meeting criteria for physical dependence requiring acute detoxification from alcohol dependence, opiate dependence or barbiturate dependence will be excluded; 7) pregnant or breastfeeding; 8) women of child-bearing potential who aren’t able to agree to the requirements specified above; 9) those who have participated in a clinical trial of an investigational drug within the past 1 month; 10) history of lithium toxicity, not due to mismanagement or overdose that required treatment.

### Dosing

Lithium carbonate will be started at 300 mg at bedtime. After 3 days, participants will be advised to increase the dosage to 600 mg, and then the dose will be titrated as clinically appropriate to maximum well-tolerated doses based on blood levels. Subsequent adjustments will be made at the discretion of the treating clinician, considering patient tolerability, response, and other medications included in treatment as part of TAU. Clinicians may increase the dose as clinically indicated and tolerated, not to exceed serum levels of 1.2 mEq/L. Clinicians will be discouraged from any dose increases that are expected to result in unacceptable tolerability. Similarly, at any time during study participation, if the 600 mg/day dose is not tolerated, the dose may be decreased to a minimum of 300 mg/day. Patients who cannot tolerate 300 mg/day of lithium will continue to receive TAU and otherwise adhere to study protocol and procedures for the duration of the trial. Treating psychiatrists will be asked to manage the patients as they would any other patient, adjusting medications as needed in response to clinical exacerbations or side effects.

### TAU

The foundation of TAU is to maintain treatment with at least one FDA approved mood stabilizer and to follow the recommendations summarized in the evidence-based stages of Texas Implementation of Medication Algorithm (TIMA) revised guidelines [43]. For all patients, TAU will require the presence of at least one FDA-approved mood stabilizer and antidepressant medications will only be prescribed in combination with a mood stabilizing drug, as described in the TIMA guidelines.

### Medication management

All participants will receive usual treatments for bipolar disorder [90] provided by either the investigator, clinical psychiatrists or psychiatry residents trained, certified, and supervised in optimal treatment practices for bipolar disorder within investigator-directed clinics. Where medication checks are being provided by non-investigator clinicians, the role of the investigator will be to monitor and/or influence/advise treatment taking place in their clinic.

Medication checks will take place every 2–4 weeks during the Open Stabilization Phase and/or as clinically indicated and weekly during the 1-month Observation phase. Medication checks during the Relapse Prevention Phase will take place every 2 months as clinically indicated. The frequency of medication checks will be adjusted as clinically appropriate at any time during the study based on the current clinical status of subjects in the study.

The CGI-S-BP for Overall Bipolar Illness, for Depression, and for Mania will be completed by the treating psychiatrist at every medication check. For subjects who are believed to be relapsing, the treating psychiatrist will complete a DSM-IV mood episode checklist for major depressive episodes, manic episodes, and mixed episodes.

### Physical and laboratory monitoring

During the baseline period, vital signs and weight will be recorded. In addition, an EKG will be required for participants over the age of 40 as clinically indicated. The EKG requirement will be waived if an EKG had been obtained within 3 months of the baseline visit and a report is available. Laboratory assessments of BUN, creatinine, electrolytes, TSH and a urine pregnancy test (if applicable) will be obtained at baseline and will be repeated based on APA guidelines for recommended lithium monitoring (see Tables 1 and 2). Drug screens will not be routinely administered; however, administration of drug screening as part of ongoing treatment is at the discretion of the treating physician at any time, after consultation with the participant.

**Table 1:** Lab monitoring schedule

	Baseline	Stabilization		O^a^	M^b^
Test	Baseline	2 weeks	12 weeks	16 weeks	Every 6 months during 24 months of additional follow-up
Vitals/Weight	X		X		X
EKG^c^	X if age > 40				
BUN	X		X		X
Creatinine	X		X		X
Pregnancy	X				
TSH	X		X		X
CBC	X				
Electrolytes	X		X		X
Lithium	X	X	X	X	X
Genetics Samples	X				

^a^Observation phase

^b^Maintenance phase

^c^If clinically indicated, as determined by the study physician

**Table 2:** Assessment schedule (time in weeks)

	B	Stabilization						O	Maintenance
	-1	0	4	8	12	14	16	Q 2 wks × 8 wks then monthly visits	Cross over to Valproic Acid for mood episode on lithium
CGI-S-BP by doctor	X	X	X	X	X	X	X	X	X
Mood episode checklist	X	X	X	X	X	X	X	X	X
Consent	X								
MINI- PLUS	X								
Demographics/course of illness	X								
HAM-A		X			X		X		X
MADRS		X	X		X		X	X	X
QIDS-SR 16	X	X	X	X	X	X	X	X	X
YMRS/CARS – M		X	X		X			X	X
Side Effects Survey	X	X	X	X	X	X	X	X	X
Q-LES-Q	X				X		X		X
IRS	X				X		X		X
MSSI	X	X	X		X		X		X
Serious Adverse Event Form	X	X	X	X	X		X	X	X

*O* observation phase, *B* baseline, *ET* Early Termination, *Mini-PLUS* Extended Mini-International Neuropsychiatric Interview, *MADRS* Montgomery Asberg Depression Rating Scale, *QIDS-SR 16* Quick Inventory of Depressive Symptomatology Self-Report, *YMRS* Young Mania Rating Scale, *CARS-M*: Clinician Administered Rating Scale for Mania, *HAM-A* Hamilton Anxiety Rating Scale, *CGI-BP* Clinical Global Impressions of Severity - Bipolar Version, *Q-LES-Q* quality of life, enjoyment, and satisfaction questionnaire, *IRS* impulsivity rating scale, *MSSI* The Modified Scale of Suicidal Ideation

### Assessments

Demographics: Age, sex, years of education, race, marital status, income, smoking status, employment history, and other demographic information will be recorded at baseline. The course followed by these patients after the study ends may offer rewarding opportunities to assess genetic correlates of long-term outcome in a sample with well-documented treatment responses. For this reason, we will also ask subjects to provide consent for future contact, as well as the names, addresses and telephone numbers of two individuals likely to know the subject’s future whereabouts, and the subject’s driver’s license number.

### Diagnosis

The Diagnostic Interview for Genetic Studies (DIGS) will be used to make DSM-IV diagnoses and to collect detailed historical clinical information. This instrument was developed to obtain very fine grain information about the symptoms and course of bipolar disorder. It has also been modified to collect retrospective medication response information. It has been validated by numerous studies and used for our collection of 4,500 subjects.

### Clinical course

Data will be collected on course of illness prior to randomization, including age of onset for bipolar disorder, number of prior episodes, past treatment response, childhood abuse (emotional, physical, sexual), medical conditions, psychoactive substance use, family history, prior lithium treatment and prior suicide attempt history, including lethality. Specifically, a history of episodes in the previous 2 years will be taken using a life chart method. This will be used as a covariate to correct for natural course in the statistical analysis.

### Symptom severity

1. Clinical Global Impressions of Severity Scale-Bipolar Version (CGI-BP) [44]. The CGI-BP is a modified version of the original CGI designed specifically for use in assessing global illness severity and/or change in patients with bipolar disorder; it assesses overall bipolar illness, depression, and mania. While the original CGI has been criticized for lack of reliability, the CGI-BP has been shown to have excellent inter-rater reliability. Not surprisingly, placebo response rates have been shown to be lower with the CGI-BP, compared to the HAM-D or MADRS in bipolar disorder. In contrast to symptom-severity scales, the CGI-BP is an integrated measure of illness severity that permits the incorporation of an assessment of function at work, social, family settings, etc. In contrast to the MADRS and HAM-D, it is not encumbered by the inability to distinguish improved somatic function (appetite and sleep) from medication-induced adverse events, which is important when studying medications that cause weight gain or somnolence.
2. Montgomery Asberg Depression Rating Scale (MADRS) [45]. A 10-item clinician-rated measure of depression will be used to examine the extent of each patient’s current depressive symptoms.
3. Young Mania Rating Scale (YMRS) [46]. An 11-item, clinician-rated measure that queries symptoms of mania.
4. Clinician Administered Rating Scale for Mania (CARS-M) [47]. The CARS-M is a reliable and valid 15-item, clinician-rated measure of mania The CARS-M incorporates a number of methodological improvements in comparison to more frequently utilized mania rating scales, such as the YMRS. For example, the CARS-M separately assesses the presence of psychotic symptoms (e.g., delusions and hallucinations). Given the overlap in symptoms assessed on the YMRS and CARS-M, an integrated version will be developed and utilized for the current study, minimizing patient burden, yet allowing full-scale scores to be derived for each measure.
5. Quick Inventory of Depressive Symptomatology Self-Report (QIDS-SR16) [48]. A 16-item self-report measure of depressive symptoms.

### Neurocognitive assessment

Neurocognition will be assessed for several reasons. Bipolar subjects with cognitive deficits may comprise a distinct group with a different disease mechanism. If this is the case, neurocognitive measures may correlate with response and serve as an endophenotype for response [49], substantially increasing the power to detect genes. Another reason has to do with side effects. Not only are such deficits part of the core bipolar phenotype, they may also be induced by medications. Cognitive slowing is a not uncommon side effect for many mood stabilizers. Testing may enable us to determine if these are markers for response, side effects or relapse, and if they are associated with specific genes.

Based on a review of previous published data on cognitive dysfunction in bipolar disorder and the heritabilities of several neurocognitive measures, our overall assessment strategy will focus on a brief measurement of three domains: attention, verbal learning, and executive function. The proposed neurocognitive battery can be administered in approximately 25 min and will consist of 5 measures (see Spreen & Strauss, 1998 for test citations):

1. Wide Range Achievement Test (3 minutes) - Third Edition, Reading (WRAT-3). The WRAT-3 Reading subtest assesses single-word reading skill.
2. WAIS-III, Digit Symbol Subtest (3 minutes). The WAIS-III Digit Symbol subtest task measures both visual scanning and graphomotor speed.
3. The Stroop Color Word Test (SCWT) (4 minutes) (Golden version). The SCWT is a commonly used executive functioning task measuring inhibitory or cognitive control.
4. Controlled Oral Word Association Test (5 minutes). The COWAT measures the ability to verbally produce words and is considered by most neuropsychologists to be a task of executive function.
5. California Verbal Learning Test (CVLT) (15 minutes). The CVLT measures the processes and strategies involved in learning and retaining verbal material. Recent data support a familial component for impaired performance on the CVLT in bipolar disease patients and their unaffected co-twins [50]. COMT genotype has been associated with performance on the CVLT in patients with bipolar I disorder [51].

### Quality of life and functioning

Quality of Life, Enjoyment, and Satisfaction Questionnaire (Q-LES-Q) [52]. Assesses subjective quality of life (i.e., physical health, subjective feelings, leisure activities and social relationships).

### Impulsivity and suicidal behavior

1. Impulsivity Rating Scale (IRS). [53] The IRS is a 7-item clinician-rated assessment of impulsivity. It uses a 0 to 3 scale to rate irritability, impatience, decision-making, distractibility, aggressiveness, response control, and delayed gratification.
2. The Modified Scale for Suicidal Ideation (MSSI*).* [54] The MSSI is an 18-item clinician-administered scale that monitors intensity of ideation, courage and competence to attempt suicide, and talk and writing about death over the past year. The first 4 items have been designated as screening items to identify those individuals whose suicide ideation is severe enough to warrant the administration of the entire scale. Each item is rated on a 0-3 point scale, and the ratings are summed to yield a total score ranging from 0 to 54.

### Side effects

Frequency and Intensity of Side Effects Ratings. A 3-item self-rated measure of medication side effects.

### Serious adverse events (SAEs)

SAEs will be systematically recorded to assess any life-threatening events or hospitalizations. An SAE is defined as any adverse drug experience occurring at any dose that results in any of the following outcomes:

- life-threatening event, including suicide attempts
- death
- hospitalization/prolongation of hospitalization
- congenital anomaly
- persistent or significant disability/incapacity
- required intervention to prevent permanent impairment/damage

### Definitions of lithium relapse and spectrum of efficacy

There is emerging consensus that compounds that possess relapse and recurrence prevention efficacy in bipolar disorder stabilize mood by different mechanisms. Compounds that stabilize mood from both above and below baseline are viewed as having bimodal efficacy, e.g., they are able to delay or prevent both depressive and manic episodes. Those that stabilize mood by delaying or preventing episodes from below baseline are described as having the ability to delay or prevent depressive episodes without causing treatment-emergent mania, whereas those that stabilize mood from above baseline are believed to delay or prevent manic episodes without causing treatment-emergent depression. To identify pharmacogenetic predictors of spectrum of efficacy by mood state, the following definitions of relapse will be used:

- Manic Relapse will be defined as patients who meet DSM-IV criteria for a manic episode but not a major depressive episode.
- Depressive Relapse will be defined as patients who meet DSM-IV criteria for a major depressive episode but not mania or hypomania. In addition, the duration of the episode must be 4 weeks instead of 2.
- Mixed Relapse will be defined as patients who meet the DSM-IV criteria for a major depressive episode and a manic episode.
- Clinician’s Judgment. In addition to the DSM-IV defined relapse criteria, a patient may be deemed a relapse if, in the judgment of the treating physician, the current regimen is not working and it is clinically necessary to make a change in medications.

### Criteria for discontinuation

Participants may be discontinued from the study at any time at investigator discretion. Specific reasons for discontinuing are 1) withdrawal of informed consent; 2) pregnancy; 3) clinically significant or serious adverse event not consistent with continuation in study, as determined by investigator or participant; and 4) suspension or termination of the study, study site, or at investigator discretion.

### Valproate crossover

Participants who are unable to tolerate lithium or fail to achieve relapse prevention on lithium will be crossed over to treatment with valproic acid in an identically designed second phase, including a 3-month stabilization phase, 1-month observation phase, and a relapse prevention phase of up to 24 months. Measurement of valproate response in a subset of subjects will make it possible to test whether SNPs associated with lithium response are also associated with valproate response.

### IPSC studies of lithium response

**Fig. 1:**
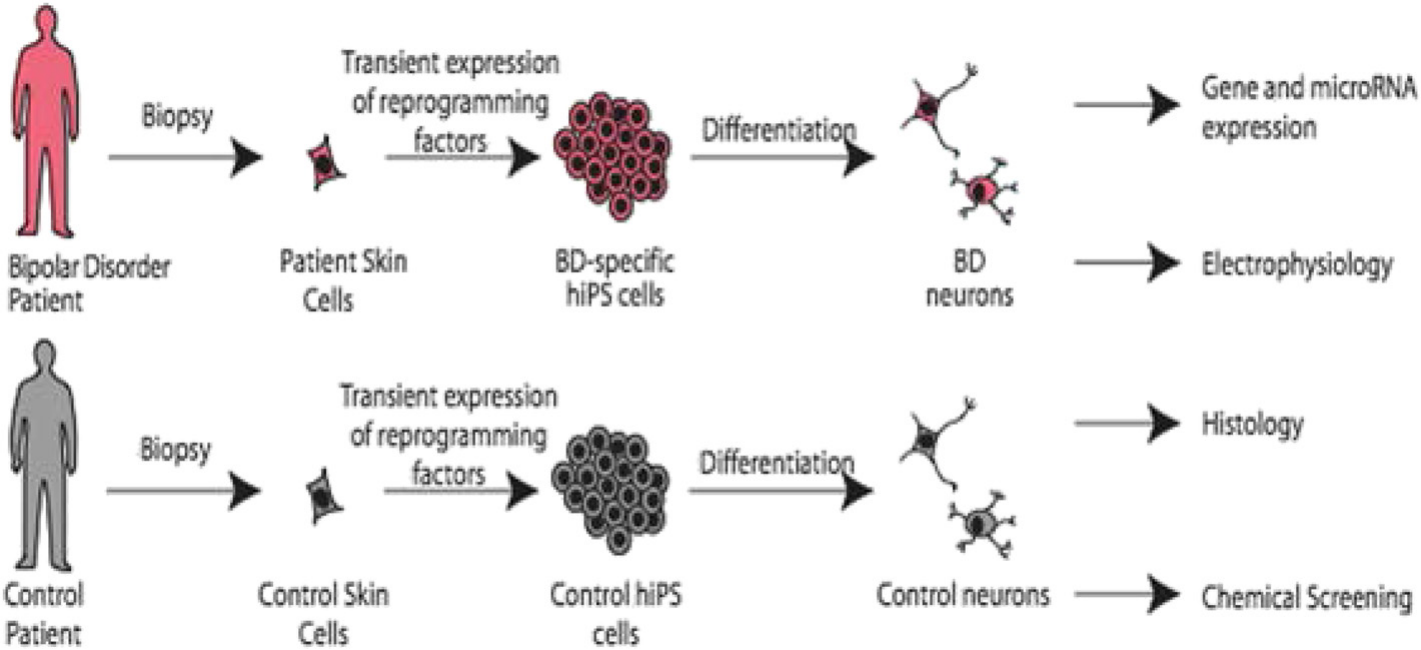
iPSC-derived neurons as a cellular model of bipolar disorder and lithium response. Fibroblasts are grown from skin biopsies taken from participating subjects. These cells are treated with a combination of four transcription factors that reprogram the cells to stem cells. These iPSCs are then treated with a series of growth factors to differentiate them into a variety of neuronal types. In this way, neurons from patients can be compared to controls or lithium responders to non-responders

We propose to create cell-based human models for lithium response in bipolar disorder by reprogramming skin fibroblasts from lithium-responsive and non-responsive bipolar disorder patients into human induced pluripotent stem cells (hiPSCs) (Fig. 1). By differentiating these disorder-specific hiPSCs into neurons, we hope to not only identify specific neuronal defects associated with bipolar disorder neurons in vitro but also elucidate the molecular and cellular mechanisms of lithium responsiveness and non-responsiveness in bipolar disorder.

### Gene expression studies will be used to identify high-priority candidate genes and to study gene networks

**Fig. 2:**
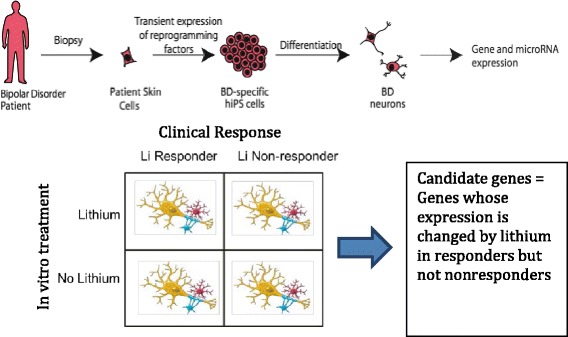
Using cellular phenotypes to identify likely lithium-responsive genes. To reduce the number of comparisons and preserve statistical power, iPSC-derived neurons will be used to identify a smaller number of genes likely to be involved in lithium’s action. iPSC-derived neurons from lithium responders and non-responders will be treated both with and without lithium. RNAseq will be conducted and genes sought whose expression changes in responders but not non-responders. These genes will comprise a limited number of a priori hypotheses for initial testing

In particular, gene expression will be studied using RNA-seq in cells from responders and non-responders treated with and without lithium, as illustrated in Fig. 2. Genes whose expression is significantly changed in response to lithium in responders but not non-responders will comprise a set of high-priority candidate genes for further study. This set of genes will then be used for detailed genotyping and sequencing studies of the DNAs from the prospective study participants. By using biology to develop a reduced set of a priori selected candidate genes, we can substantially reduce the number of statistical tests and preserve the statistical power of our sample. This strategy assumes that some of the same genes displaying differential change in expression also contain the variants affecting lithium response. Candidate genes may also be selected from other sources such as published literature or results from the ConLiGen consortium, animal or cellular studies.

The gene expression data will also be used to develop gene expression networks that can then be compared between the four conditions. It is likely that, more than specific genes, it is changes in the functional modules or groups of genes that are most robustly changed in response to lithium. Such a network-based approach may also be more informative regarding the biology that is altered by lithium and different in responders.

### Genotyping and genetic data

Genotyping will be completed using PsychChip, which was developed on an Illumina platform for the Psychiatric Genomics Consortium (PGC). This chip includes the 50,000 most significant SNPs from several psychiatric disorders, as well as 250 K SNPs exome and framework GWAS content. We will use PLINK (http://pngu.mgh.harvard.edu/purcell/plink/) to evaluate and clean the genetic data received from TGen for 1) genotypic completeness, 2) average SNP heterozygosity, and 3) average genotyping quality score. For each SNP we will review 1) genotypic completeness, 2) average SNP heterozygosity, 3) average genotypic quality score, 4) minor allele frequency (MAF), and 5) p-values for tests of deviation from Hardy-Weinberg equilibrium. After generating a filtered dataset, we will examine Q-Q plots of association statistics with case-control status defined by good versus poor responders in order to look for systematic deviations from expectation that may further reflect artefacts in the data. Through these procedures, we will generate a final, cleaned dataset for the downstream analyses. Notable findings will all be reviewed in further detail as a final quality control check to confirm that they are not due to artefacts of low MAF, low genotyping completeness, batch or laboratory effects, or population stratification. In addition, we will visually examine the actual intensity plots of the individual best SNPs to confirm the quality of the genotype calls and ensure there is no evidence of systematic error that might lead to spurious results.

### Statistics

For the analysis of the Prospective Study, we will examine the outcome for lithium response as a time to event, where the event is defined as clinical relapse as defined above. For this we will use Cox Proportional Hazards models of the form:

$$ \log\ \mathrm{h}\left(\mathrm{t}\right) = \upalpha \left(\mathrm{t}\right) + {\upbeta}_1{\mathrm{X}}_1 + \dots + {\upbeta}_{\mathrm{k}}{\mathrm{X}}_{\mathrm{k}} $$

where h(t) is the hazard of relapse at time (t) and the time axis is defined as the time from stabilization on drug to either clinical relapse, censoring due to loss to follow-up or discontinuation of study medication, or the end of the follow-up period. This model is semi-parametric in that the baseline hazard of relapse can take any form, but the covariates enter the model linearly and are assumed to have constant relative hazard over the entire time period. This assumption of constant relative hazard over time can be evaluated (and remedied if needed) by including in the model interactions terms between the covariate of interest and time. Building on this framework, we will test in separate models whether the SNPs identified in the ConLIGen Retrospective Study are significantly associated with the relative hazard of clinical relapse. We will include in the models covariates for age, sex, clinical site, and up to 10 quantitative indices of ancestry (if preliminary analyses of the genetic data suggest evidence of population stratification in the sample) to control for the potential confounding effects on these associations. We will also be able to include a covariate for an indicator variable capturing whether the patient entered the prospective study already on lithium as allowed by the protocol in order to control for this as well as to test whether associations differ for these versus other patients.

We will also examine repeated quantitative measures of depressive or manic symptoms taken at regular intervals over the period of follow-up. This will address the number of symptoms subjects continued to suffer even when they were considered in remission. For the repeated measures analyses, we will use longitudinal data methods to associate SNPs with differences in symptom measures over time. In particular, we will use linear regression models with general estimating equations (GEE) to account for the correlation engendered by repeated measures on the same participants. We will explore the repeated measures data to diagnose the nature of this correlation and then treat it as a nuisance variable in the model. We will then test whether the SNPs identified in the Retrospective Study or other retrospective datasets (ConLiGen) are significantly associated with differences in mean scores on the measures of depressive or manic symptoms, controlling for time of assessment, age, sex, clinical site, and the same quantitative indices of ancestry as the in the Cox model if deemed necessary. We will further explore for possible interactions between the SNP and time to test whether the effect of the SNP on depressive or manic symptoms changes over time.

To determine whether the effects of SNPs found to be associated with response in these analyses are specific to lithium treatment, we will use two strategies. First, in the time to event and repeated measures analyses described above, we will examine whether the inclusion of a covariate describing prior disease episode frequency affects the estimated association between SNPs and clinical relapse and/or symptom differences. If the estimated association is mitigated, this will suggest that the SNPs are associated with the natural course of disease in general. If, however, the estimated association persists, this will suggest that the association is specific to lithium responsiveness. To further confirm this possibility, we will examine the data from the Depakote arm of the Prospective Study. We will test whether the identified SNPs predict clinical relapse or symptom differences among patients who are crossed over to Depakote. If they are not associated, this will suggest the variants are associated specifically with lithium responsiveness.

### Power

**Fig. 3:**
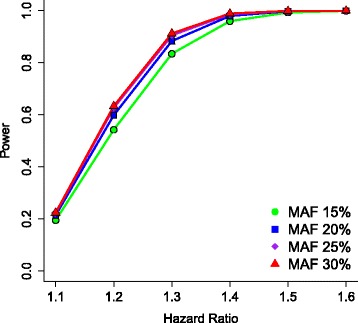
Power of survival analysis as a function of hazard ratio and allele frequency for 700 subjects. Power analyses indicate that a sample of 700 subjects will have about 80 % power to detect a hazard ratio of 1.4

We calculated the power for the survival analysis of the Prospective Study following the methods of Lakatos [55] based on a proportional hazards model and assuming a nominal significance alpha of 0.05. We further assumed a dominant model to simplify the calculations, but note that this model typically provides a conservative estimate of power compared to a log additive model, which is what we plan to assume in the analyses. Finally, we assumed 2 years of follow-up with a relapse rate of 50 % among participants with the wild type genotype. Figure 3 below shows the power of our sample to identify a significant association under these conditions for an estimated sample of 700 patients in the Prospective Study for various hazard ratios and minor allele frequencies.

As described above, we plan a two-stage analysis in which a small number of a priori hypotheses will be tested in stage 1 so as to preserve power. SNPs to be tested in this stage will be selected from our collaborating retrospective lithium study, ConLiGen, significant SNPs from our retrospective sample, SNPs reported in the literature and those selected from expression studies of iPSC-derived neurons. In a second stage of analysis, a full GWAS will be conducted as an exploratory analysis to discover novel variants that may be replicated in other samples.

### Ethics

This international study will be conducted in nine clinical centers in the US, one in Canada, and one in Norway. The study sponsors are the National Institutes of Mental Health and General Medical Sciences in the US (MH92758), The Western Norway Regional Health Authority and the Canadian Institutes of Health Research (#64410). The study protocol has been approved by both the local IRB’s at all US sites and in Canada, as well as the Ethics Committee and Health Authorities in Norway. The investigator is responsible for ensuring that no patient is subject to any study-related examination or activity before that patient has given written informed consent after the receipt of detailed information. The investigator will inform the patient of the aims, methods, anticipated benefits and potential hazards of the study, including any discomfort it may entail. This information will be summarized in integrated patient information and consent sheets. All sites participating in the collection of fibroblasts have obtained an additional IRB approval for the iPSC studies and all additional informed consents regarding this procedure have been obtained.” The study has been registered on the EudraCT database (EudraCT number 2010-023740-32) and at ClinicalTrials.gov (NCT01272531). The trial will be conducted in accordance with the latest version of the Declaration of Helsinki and the International Conference on Harmonization (ICH) - Good Clinical Practice (GCP) guidelines for clinical trials. The ethics committees are as follows:

- Regional committees for medical and health research ethics: REC South East, University of Bergen
- The University Hospitals Institutional Review Board (IRB), University Hospitals, Cleveland, OH
- Capital Health Research Ethics Board, Center for Human Research, Dalhousie University
- Human Subjects Office/Institutional Research Board, University of Iowa
- Office of Research Compliance – Indiana University
- Office of Human Subjects Research Institutional Review Boards, Johns Hopkins University
- Mayo Clinic Institutional Research Board
- Human Research Protection Program (HRPP), University of Michigan
- University of Pennsylvania, Institutional Research Board
- BSD IRB Committee C, University of Chicago
- Human Research Protections Program, University of California, San Diego

## Discussion

Based upon the well-established clinical efficacy of lithium in the treatment of bipolar disorder, we present here the study protocol of a large, multicenter prospective study designed to identify genetic predictors of good lithium response. The study is an 11-site open non-randomized trial of 700 Bipolar I patients treated with lithium monotherapy and followed for up 2 years. The long-range goal of the study is to better understand lithium’s mechanism of action and to develop a possible DNA test panel that will aid physicians in medication selection.

During the study development, the following factors were considered:

1. Prospective vs retrospective. Almost all previous studies of lithium response have been done using standardized chart review and retrospective response data, which has the advantage of surveying the subject’s entire lifetime experience on lithium. However, it suffers the serious disadvantages of poor recall, state-related recall, lack of monotherapy (in general), qualitative instead of quantitative data and lack of observer ratings. For these reasons, we chose a prospective design. Though the much greater effort and expense will only allow for smaller sample sizes, we deemed the accuracy and superiority of the data to be the more important factors. This sample may serve as a “gold standard” sample for future studies, especially for replication of results.
2. How many mood stabilizers to study? We considered a design where subjects would be randomized between lithium and two other mood stabilizers such as lamotrigine or valproate. Though this design would have given us invaluable comparative information, we chose instead to have a larger sample size and better statistical power for one drug, lithium. The crossover of lithium failures to valproate provides some opportunity for comparison.
3. How to distinguish gene effects from natural course? If a genetic variant shows association to response, how do we know that this reflects the subject’s response to lithium vs. simply the natural course of the illness? Specifically, how do we know that a SNP is associated to lithium response or simply rapid cycling and is unrelated to drug? The scientifically ideal design would be to include a placebo arm as a control for natural course. However, given the severity of bipolar disorder, we deemed it neither ethical nor feasible to include such an arm. Instead, we have employed a detailed collection of information about lifetime cycling to covary for this effect. Of note, in the Perlis et al. study of lithium response in the STEP-BD sample, a similar inclusion of rapid cycling as a covariate had no effect on the results [42].
4. Monotherapy. Though perhaps a therapeutic ideal, monotherapy using any drug is not usually achieved in the management of bipolar disorder. However, if we allowed multiple drugs, then we would remain unclear as to which drug was driving the response and to which drug a genetic variant is associated. For these reasons, we chose to require monotherapy, though it excludes partial responders and the generalizability.
5. Acute vs prophylactic response. A shorter and faster study might have focused on the acute response to lithium in treatment of depression, mania or mixed states. However, the most widely used application for lithium is prophylaxis, and by studying relapse prevention we focus on the greatest clinical utility. Most patients also enter the study on other medications and the acute stabilization period is confounded by these other medications and the changes in medications during this period
6. Duration of follow up. Twenty-four months of follow up was selected because the STEP-BD study showed that, at 2 years, about 50 % of treated bipolar I patients had relapsed. Two years was therefore selected to maximize power.

In summary, only a limited investigation has been made of this area. Generally only a few genes have been tested and samples are largely retrospective. STEP-BD is the only truly prospective study, yet only about 200 subjects achieved monotherapy; other results are confounded by concomitant meds. There is a need for larger, better characterized samples and a more systematic approach to this area.
